# Health Beneficial Properties of Grapevine Seed Extract and Its Influence on Selected Biochemical Markers in the Blood, Liver and Kidneys of *Rattus norvegicus*

**DOI:** 10.3390/molecules26072099

**Published:** 2021-04-06

**Authors:** Lenka Sochorova, Mojmir Baron, Katerina Dadakova, Tomas Kasparovsky, Jiri Sochor

**Affiliations:** 1 Department of Viticulture and Enology, Faculty of Horticulture, Mendel University in Brno, Valtická 337, 69144 Lednice, Czech Republic; tomaskova.l.9@gmail.com (L.S.); mojmir.baron@mendelu.cz (M.B.); 2 Department of Biochemistry, Faculty of Science, Masaryk University, Kotlarska 2, 61137 Brno, Czech Republic; k.dadakova@mail.muni.cz (K.D.); tkasp@sci.muni.cz (T.K.)

**Keywords:** grape seed extract, antioxidants, cadmium, *rattus norvegicus*, biochemical markers, protective effect

## Abstract

Cadmium (Cd) is a heavy metal that occurs in all areas of the environment, including the food chain. In the body, it causes oxidative stress by producing free radicals that are harmful to the cells. Grape seed extract (GSE) contains a wide range of biologically active components that help to neutralize the adverse effects of free radicals. In this study, the effects of GSE prepared form semi-resistant grapevine cultivar Cerason, which is rich in phenolics, on biochemical markers of brown rats exposed to the effects of cadmium were monitored. GSE increased the plasma antioxidant activity and, in the kidneys and the liver, Cd content was significantly lowered by GSE co-administration. Accordingly, the increase in creatinine content and alanine aminotransferase activity and the decrease of catalase and superoxide dismutase activities caused by cadmium were slowed down by GSE co-administration. The results of this work reveal that grape seed extract offers a protective effect against the intake of heavy metals into the organism.

## 1. Introduction

Cadmium (Cd) is a heavy metal that occurs in all areas of the environment; it is part of the Earth’s crust, is contained in water, accumulates in plants, and is present in organisms. In addition to the food chain (in terms of plants, most cereals, root crops, and vegetables are sources, while among animal organs, primarily the liver and kidneys are a source), an important source of cadmium for humans is cigarette smoke, exhalates from various types of combustion, metallurgical industries, and some electrical installations [1,2].

In the body, cadmium binds predominantly to metallothionein, albumin, and other plasma proteins in the blood. Cadmium is a dangerous toxic metal that causes oxidative stress by producing free radicals that are harmful to the cells. Free radicals can damage proteins, lipids, enzymes, and DNA, and therefore must be neutralized by antioxidants before entering the cells [3].

If not neutralized, Cd causes the disintegration of red blood cells [4], accumulates in the organism—especially in the liver and kidneys—and, due to its similarity to zinc, which is an essential element, it easily enters enzymatic reactions and participates in biochemical processes [5]. It is excreted only very slowly and with difficulty from the organism [6].

It is well-known that antioxidants can have a protective effect against oxidative stress and heavy metal damage, and this experimental work was based on this fact. Grape seed extract (GSE) contains a wide range of biologically active components in its matrix [7] that help to neutralize the adverse effects of free radicals. GSE protects cells from damage by regulating cellular oxidative damage, reducing organ damage, improving the balance between oxidants and antioxidants, and reducing the release of inflammatory mediators. It also has anti-inflammatory, antiapototic, and pro-proliferative effects [8]. The high content of condensed flavan-3-ol or procyanidins, which are under investigation as an alternative method of treatment for serious diseases, is particularly significant [9,10].

However, the content and composition of grape seed phenolics, as well as the antioxidant activity of the seed extract, can be influenced by grapevine cultivar, rootstock, climatic conditions, or growing techniques [11,12,13] and, therefore, antioxidant properties as well as protective effects of a concrete extract cannot be easily predicted. The aim of this work was to study the protective effect of GSE prepared from semi-resistant grapevine cultivar Cerason on biochemical markers in the blood, plasma, liver, and kidneys of brown rats that had been exposed to the effects of cadmium.

## 2. Results and Discussion

### 2.1. Antioxidant Activity and Phytochemical Composition of Grape Seed Extract

The grape seed extract prepared from the semi-resistant variety Cerason was tested for antioxidant activity, total polyphenolic content, and selected individual antioxidants.

Antioxidant activity was determined spectrophotometrically using four methods based on different principles (DPPH^●^, ABTS^●+^, FRAP, FR) and the total polyphenolic compounds were determined with the Folin–Ciocalteu method. Depending on the method used, the antioxidant activity ranged from 7 to 31 mg/g gallic acid equivalent (GAE) and the total polyphenolic content was 9.3 mg/g GAE (Table 1).

Using HPLC with MS detection, the content of the selected antioxidant components was determined. Attention was focused on the content of eight flavonoids (apigenin, astragalin, hyperoside, isorhamnetin, kaempferol, myricetin, quercitrin, and rutin) and two procyanidins (procyanidin A2 and procyanidin B1). The most represented compound of the monitored antioxidants was procyanidin A2 (average content was 359 µg/g). The least represented compound was kaempferol, which had an average content of 0.21 µg/g (Table 2).

The results of this part of the experiment pointed to the high antioxidant potential in grape seeds.

### 2.2. In Vivo Determination of the Grape Seed Extract Effect in the Rats

#### 2.2.1. Determination of Cadmium Content in Blood, Liver, and Kidneys

Cadmium is the most prevalent toxic metal in feeds. After oral administration, it is absorbed from the intestine, transported to the liver, and conjugated to thiol groups of metallothionein (MT). Apart from this, Cd can also be bound to albumin, or other proteins in the blood [14]. Subsequently, Cd accumulates in the body, is excreted only slowly from the body via the urine and feces and has a biological half-life of about 20 years. The largest amount (about a third) of cadmium is found in the kidney cortex. In the blood, most cadmium (about 90%) is present in the red blood cells. It is toxic to a wide range of organs and the main targets are the kidneys, bones, and lungs [15,16,17]. GSE has been reported to lower cadmium concentrations in the red blood cells and in the kidneys, whereas its content in the liver has been reported to increase [14].

**Figure 1:**
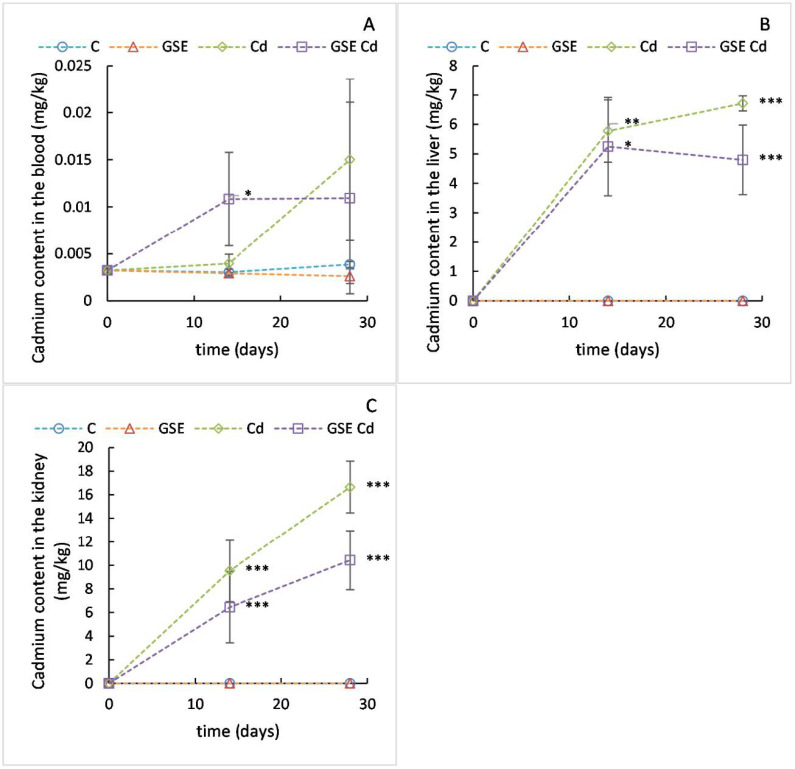
Cadmium content values in the blood (**A**), liver (**B**), and kidneys (**C**) for the control variant (blue line with circles), the variant with grape seed extract (GSE) addition (orange line with triangles), the variant with cadmium (Cd) addition (green line with diamonds), and the variant with both GSE and Cd additions (violet line with squares) on days 0, 14, and 28 of monitoring. Data are presented as means ± sd from three replicates. Data were analyzed by one-way ANOVA (each time point separately) and differences from control are marked: * indicates *p* ≤ 0.05, ** indicates *p* ≤ 0.01, *** indicates *p* ≤ 0.001.

The results for the blood cadmium content in this study are shown in Figure 1A. At the beginning of the experiment, 3.2 µg/kg of cadmium were found in the blood of the animals. During the experiment, after 14 days, the mean cadmium value in the group with the addition of GSE and cadmium was significantly higher than in other groups (10.8 µg/kg, *p* = 0.023). After 28 days, the variability of the data was too high to observe a statistical significance.

At the beginning of the experiment, no cadmium was detected in the livers (Figure 1B) of the experimental animals. Accordingly, no cadmium was detected in the livers of the animals from the control and GSE groups during the experiment. On the other hand, after 14 days of the experiment, the mean cadmium value in the group with the addition of cadmium was 5.8 mg/kg and in the group with the addition of cadmium and GSE, the mean value was 5.2 mg/kg. Both these values were significantly different from the control group (*p* = 0.002 and *p* = 0.018 for the cadmium-only group and the group with cadmium and GSE, respectively). After 28 days, the mean value in the group with the addition of cadmium had increased to 6.7 mg/kg, which differed significantly from the cadmium level in the group with the addition of cadmium and GSE, that had decreased slightly to 4.8 mg/kg (*p* = 0.008).

At the beginning of the experiment, no cadmium was detected in the kidneys (Figure 1C) of the experimental animals. Accordingly, no cadmium was detected in the kidneys of the animals from the control and GSE groups during the experiment. On the other hand, after 14 days, the mean cadmium value in the group with the addition of cadmium was 9.5 mg/kg and in the group with the addition of cadmium and GSE, the mean value was 6.5 mg/kg. Both these values were significantly different from the control group (*p* < 0.001 for both groups). After 28 days, the mean value in the group with the addition of cadmium had increased to 16.6 mg/kg, which differed significantly from the cadmium level in the group with the addition of cadmium and GSE, which had increased only slightly to 10.4 mg/kg (*p* = 0.009).

Thus, in the kidneys and in the liver, Cd content was significantly lowered by GSE administration. GSE contains phenolic compounds that can chelate Cd, thus reducing the accumulation of Cd. The formation of these chelates probably resulted in a reduced ability of the proteins to bind Cd, which thus could be eliminated more effectively from the body [18].

#### 2.2.2. Determination of Creatinine and Urea Contents

With respect to the cadmium accumulation in the kidneys, the kidney is one of the most important target organs for Cd-induced toxicity. Therefore, the kidney markers creatinine and urea were determined in the plasma. The determination of creatinine is a good indicator of glomerular filtration and is used mainly to monitor the course of kidney disease. The relation between creatinine concentration and glomerular filtration is hyperbolic; as glomerular filtration decreases, creatinine secretion also decreases [19]. Increased intake of the heavy metal cadmium leads to kidney damage, and therefore reduced creatinine secretion efficiency. As a result, an elevated blood creatinine level indicates a renal disorder [20].

**Figure 2:**
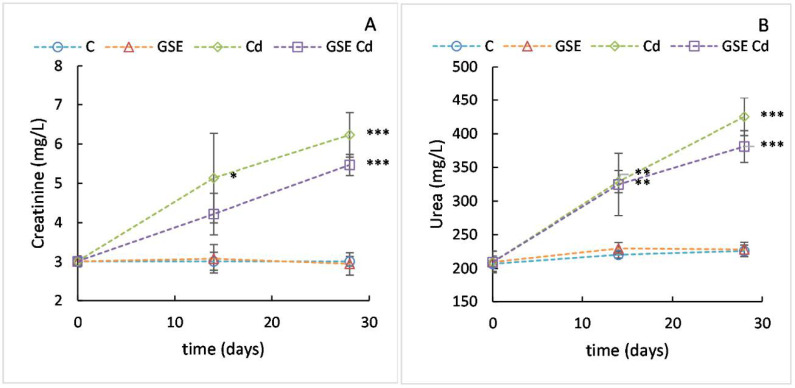
Plasma creatinine (**A**) and urea (**B**) values for the control variant (blue line with circles), the variant with GSE addition (orange line with triangles), the variant with Cd addition (green line with diamonds), and the variant with both GSE and Cd addition (violet line with squares) on days 0, 14, and 28 of monitoring. Data are presented as means ± sd from three replicates. Data were analyzed by one-way ANOVA (each time point separately) and differences from control are marked: * indicates *p* ≤ 0.05, ** indicates *p* ≤ 0.01, *** indicates *p* ≤ 0.001.

In our study, the levels of creatinine were increased by Cd, in concordance with previously published results [21]. The mean creatinine content value (Figure 2A) in the experimental animals at the beginning of the experiment was 3.0 mg/L. In the group receiving cadmium, there was a significant increase in the monitored values to 5.1 mg/L (after 14 days, *p* = 0.015) and to 6.2 mg/L (after 28 days, *p* < 0.001). In the group for which GSE was co-administered with cadmium, the increase in this marker was lower (4.2 mg/L after 14 days and 5.5 mg/L after 28 days) and statistically significant only after 28 days, when compared to control (*p* < 0.001). Thus, the increase in creatinine concentration was slowed down by GSE co-administration, suggesting a reduction of the toxic effect caused by cadmium. In the group receiving GSE only, there were normal values.

Urea is quantitatively the most important degrading product of amino acids and proteins. It is formed in the liver from ammonia released by deamination reactions during amino acid metabolism. It is excreted from the organism mainly by the kidneys by glomerular filtration and tubular resorption [22]. In the case of renal diseases, the rate of urea production exceeds the rate of its clearance, which results in an increase in blood urea level. Moreover, cadmium inhibits the incorporation of amino acids into proteins, causing uremia [21]. On the other hand, when liver function fails, urea synthesis decreases and therefore also its concentration in plasma [20]. Thus, blood urea concentration depends on dietary protein content, renal excretion, and the metabolic function of the liver.

In our study, the levels of urea were increased by Cd, in concordance with previously published results [21]. The mean urea content value (Figure 2B) in the experimental animals at the beginning of the experiment was 207 mg/L. In the group receiving cadmium, there was a significant increase in the monitored values to 329 mg/L (after 14 days) and 425 mg/L (after 28 days). In the group for which GSE was co-administered with cadmium, the increase in this marker was only slightly lower—325 mg/L after 14 days and 381 mg/L after 28 days. Both the groups receiving only cadmium and cadmium together with GSE differed significantly from the control group after 14 (*p* = 0.001 and *p* = 0.002, respectively) and 28 days of the experiment (*p* < 0.001 for both). In the group receiving GSE only, normal values were found.

#### 2.2.3. Determination of Plasma Antioxidant Activity

Cadmium induces a range of effects connected primarily to oxidative stress. As Cd is not redox active, it does not generate reactive oxygen species directly. Nevertheless, their production after Cd exposure has been reported in many studies and reviewed by Cuypers et al. [23]. The mechanisms leading to redox disturbance during Cd exposure include the depletion of reduced glutathione caused by the affinity of Cd to thiol groups, the replacement of Fe by Cd in proteins leading to increased concentrations of redox-active free Fe, mitochondrial dysfunction, and induction of NADPH oxidases, which catalyze the formation of superoxide radicals [23]. During oxidative stress, antioxidative compounds like phenolics can terminate the production of new reactive species via their conversion to stable radical forms. Therefore, plant extracts rich in phenolics can provide protective effects against Cd or Cd-containing compounds [24].

**Figure 3:**
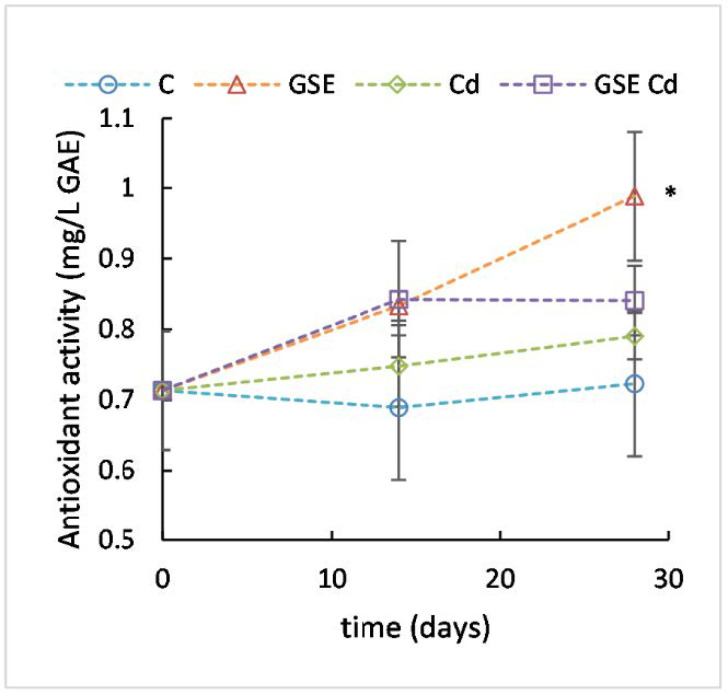
Plasma antioxidant activity for the control variant (blue line with circles), the variant with GSE addition (orange line with triangles), the variant with Cd addition (green line with diamonds), and the variant with both GSE and Cd addition (violet line with squares) on days 0, 14, and 28 of monitoring. Data are presented as means ± sd from three replicates. Data were analyzed by one-way ANOVA (each time point separately) and differences from control are marked: * indicates *p* ≤ 0.05.

In this study, the mean plasma antioxidant activity value (Figure 3) in the experimental animals at the beginning of the experiment was 0.71 mg/L. A slight increase was observed in all experimental groups as compared to the control group. However, the only significant difference was that between GSE-treated group and the control group after 28 days (*p* = 0.012), confirming a positive effect of GSE on plasma antioxidant activity. Previously reported results are ambiguous; sometimes, an increase in plasma antioxidant potential caused by GSE was found [25] and sometimes not [14].

#### 2.2.4. Determination of ALT and AST Activities

Oxidative stress is related to a loss of functional integrity of membrane architecture caused by increased lipid peroxidation. In turn, the increased permeability of the cytoplasmic membrane leads to the leakage of hepatic marker enzymes (ALT and AST) into blood [14]. Indeed, ALT and AST are diagnostically significant even in limited liver damage due to intoxication and their increase in serum caused by cadmium has been reported earlier [14] and was confirmed by this study.

**Figure 4:**
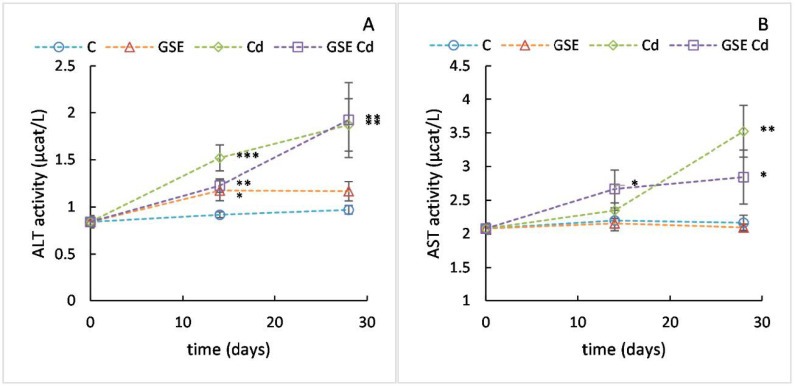
Plasma ALT (**A**) and AST (**B**) content values for the control variant (blue line with circles), the variant with GSE addition (orange line with triangles), the variant with Cd addition (green line with diamonds), and the variant with both GSE and Cd addition (violet line with squares) on days 0, 14, and 28 of monitoring. Data are presented as means ± sd from three replicates. Data were analyzed by one-way ANOVA (each time point separately) and differences from control are marked: * indicates *p* ≤ 0.05, ** indicates *p* ≤ 0.01, *** indicates *p* ≤ 0.001.

The mean ALT activity value (Figure 4A) in the experimental animals at the beginning of the experiment was 0.84 µkat/L. In the group receiving cadmium, a significant increase in the monitored values was recorded to an average of 1.52 µkat/L after 14 days (*p* < 0.001), and 1.87 µkat/L after 28 days (*p* = 0.004). In the group for which GSE was co-administered with cadmium, the increase of this marker was significantly slower than in the group receiving only cadmium (1.23 µkat/L after 14 days and 1.92 µkat/L after 28 days). After 14 days of the experiment, this group was different from both the control group (*p* = 0.009) and the cadmium group (*p* = 0.026), whereas, after 28 days, it differed significantly only from the control group (*p* = 0.003). In the group receiving GSE only, the values also increased to 1.17 µkat/L after 14 days. This value differed significantly from the control group (*p* = 0.024) and was comparable to the value of the group receiving both GSE and cadmium. After 28 days, the values of the GSE-only group resembled the control group.

The mean AST content value (Figure 4B) in the experimental animals at the beginning of the experiment was 2.07 µkat/L. In the group receiving cadmium, a statistically significant increase in the observed values was recorded only after 28 days of the experiment (3.52 µkat/L, *p* = 0.001). Surprisingly, in the group for which GSE was co-administered with cadmium, the increase of this marker was quicker and significantly different from the control group after both 14 days and 28 days (2.67 and 2.84 µkat/L, respectively, *p* = 0.043 for both), although the value was slightly lower than that of the cadmium-only group after 28 days. The GSE-only variant had normal values.

In our experiment, GSE administration was able to slow down the increase of ALT activity. This effect corresponds to the results reported previously [14] and may be caused by scavenging lipid peroxides via hydrogen transfer from a phenolic compound to the free radical-containing lipid peroxide. On the other hand, in the longer term, this effect was low. The increase of AST activity caused by cadmium was rather accelerated by GSE administration. However, in the longer term there was a slight decrease in AST activity. Thus, GSE can partially compensate for the toxic effect of Cd on cell membranes.

#### 2.2.5. Determination of SOD and CAT Activities in the Liver

In addition to direct participation in oxidative reactions, cadmium inhibits the activity of antioxidant enzymes such as SOD and CAT, enzymes able to detoxify superoxide radicals and hydrogen peroxide, respectively [14]. SOD is an enzyme that effectively quenches the superoxide radical and converts it into the less toxic hydrogen peroxide, which can be further decomposed by catalase or peroxidase [26]. CAT is an enzyme that is found in almost all living organisms that are exposed to oxygen. It acts as a catalyst for the decomposition of hydrogen peroxide into water and oxygen [27]. It has one of the highest conversion numbers among enzymes [28] and, as an antioxidant enzyme, it is one of the protective mechanisms that leads to the maintenance of balance in the human body [29]. The mechanism of Cd-induced decrease in enzyme activities is probably direct binding of Cd to the active sites of the enzymes [30].

**Figure 5:**
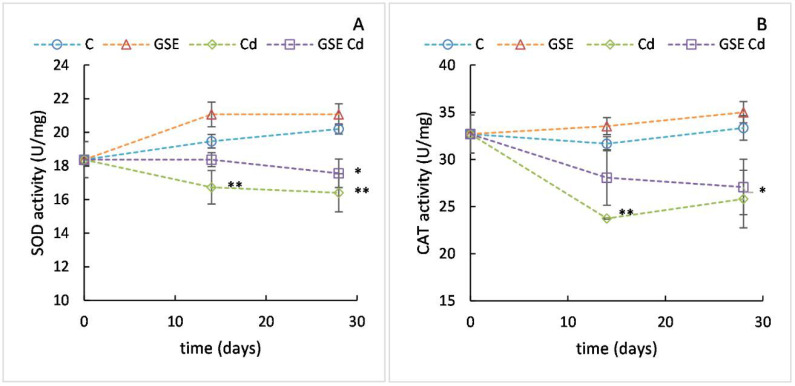
SOD (**A**) and CAT (**B**) activity values in the liver for the control variant (blue line with circles), the variant with GSE addition (orange line with triangles), the variant with Cd addition (green line with diamonds), and the variant with both GSE and Cd addition (violet line with squares) on days 0, 14, and 28 of monitoring. Data are presented as means ± sd from three replicates and expressed in U/mg of the protein. Data were analyzed by one-way ANOVA (each time point separately) and differences from control are marked: * indicates *p* ≤ 0.05, ** indicates *p* ≤ 0.01.

The decreases in SOD and CAT activities were also observed in this study. At the beginning of the experiment, the mean SOD enzyme activity in the liver (Figure 5A) of the experimental animals was 18.4 U/mg of the protein. After 14 days of the experiment, SOD activity was significantly higher in the group receiving GSE (21.1 U/mg) than in both the cadmium-receiving groups (*p* < 0.001 and *p* = 0.009 for the cadmium-only and cadmium with GSE groups, respectively), but only in the group receiving cadmium alone was SOD activity significantly lower (16.7 U/mg) than in the control group (*p* = 0.005). After 28 days of the experiment, both the cadmium-receiving groups differed significantly from both the control group (16.4 U/mg, *p* = 0.002 and 17.6 U/mg, *p* = 0.023 for cadmium only and cadmium with GSE, respectively) and the group treated with GSE only (*p* < 0.001 and *p* = 0.005 for cadmium only and cadmium with GSE, respectively).

At the beginning of the experiment, the mean CAT enzyme activity in the liver (Figure 5B) was 32.7 U/mg of the protein. After 14 days of the experiment, CAT activity was significantly higher in the group receiving GSE (33.5 U/mg) than in both the cadmium-receiving groups (*p* = 0.002 and *p* = 0.031 for the cadmium-only and cadmium with GSE groups, respectively), but only in the group receiving cadmium alone was CAT activity significantly lower (23.8 U/mg) than in the control group (*p* = 0.005). However, after 28 days, CAT activity in the group receiving GSE together with cadmium decreased further to 27.1 U/mg, reached the values of the cadmium-only group (25.8 U/mg), and differed significantly from the control group (*p* = 0.033). The group treated with GSE only differed significantly from both the cadmium groups (*p* = 0.026 and *p* = 0.012 for cadmium only and cadmium with GSE, respectively).

Interestingly, the decreases caused by cadmium were slowed down by GSE co-administration. For CAT, these effects of GSE have been observed previously [14]. The chelating effect of the phenolic compounds contained in GSE is likely to reduce the ability of cadmium to bind to the active site of the enzyme.

#### 2.2.6. Determination of MT Content

Cadmium is not subject to any direct metabolic conversions, but in all tissues it is mainly bound to metallothionein (MT). MT belongs to a group of intracellular, low-molecular-weight, cysteine-rich proteins with a molecular weight of 6–10 kDa. The primary role of MT in the organism includes maintaining zinc homeostasis, detoxifying heavy metals, and protecting against oxidative stress. MT expression is inducible by many exogenous and endogenous factors such as UV radiation, heavy metals, stress hormones, free oxygen radicals, mediators of inflammation, infection, trauma, and oncogenesis [31,32]. MT is largely synthesized in the liver and kidney and released into blood. Even though MT is responsible for cadmium accumulation in tissues by lowering its excretion, MT protects against acute as well as chronic Cd-induced toxicity [33]. In our study, the MT content was monitored in the blood, liver, and kidney.

**Figure 6:**
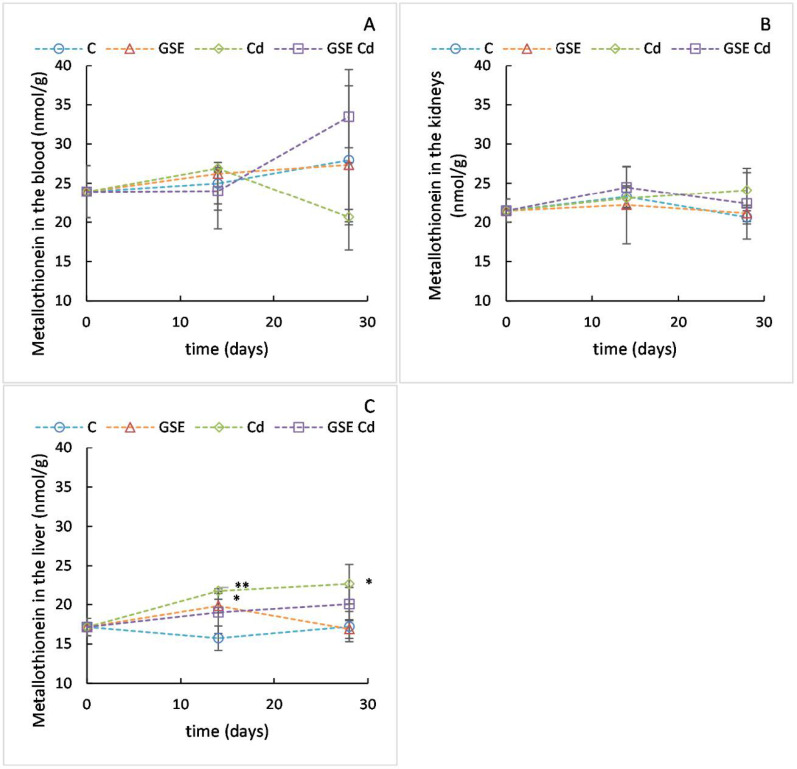
MT content values in blood (**A**), kidneys (**B**), and liver (**C**) for the control variant (blue line with circles), the variant with GSE addition (orange line with triangles), the variant with Cd addition (green line with diamonds), and the variant with both GSE and Cd addition (violet line with squares) on days 0, 14, and 28 of monitoring. Data are presented as means ± sd from three replicates. Data were analyzed by one-way ANOVA (each time point separately) and differences from control are marked: * indicates *p* ≤ 0.05, ** indicates *p* ≤ 0.01.

The mean MT content value in the blood (Figure 6A) of the experimental animals at the beginning of the experiment was 23.9 nmol/g. After 14 days, the changes were minimal and, after 28 days, large variability between the replicates was observed, causing statistical inconclusiveness (*p* = 0.948). The mean MT content value for the kidneys (Figure 6B) of the experimental animals at the beginning of the experiment was 21.46 nmol/g. The values did not change significantly in any experimental group during the experiment (*p* = 0.938). The mean MT content value in the liver (Figure 6C) of the experimental animals at the beginning of the experiment was 17.15 nmol/g. After 14 days, the MT content increased slightly in all experimental groups and the differences from the control group were significant for the Cd-only group and the GSE-only group (*p* = 0.008 and *p* = 0.046, respectively). After 28 days, the MT content in the GSE group returned to the value of the control group, whereas the MT content in the Cd group stayed increased and differed from both the control and the GSE group (*p* = 0.046 and *p* = 0.035, respectively). In the group receiving GSE together with cadmium, the MT content did not differ significantly from control—which was in concordance with the lower cadmium content found in the liver of this group—when compared to the Cd-only group (the difference was significant after 28 days of the experiment).

Altogether, statistically significant changes in the MT content caused by Cd were only observed in the liver. Previously, an MT increase in the blood of experimental animals receiving cadmium was reported [34]. In this case, the results suggest that the detoxification of Cd via MT may rather occur in the liver.

## 3. Materials and Methods

### 3.1. Experiment Design

Brown rats of the *Wistar* genus (eight weeks old, 235 ± 3 g) were divided into four groups of six individuals: A) a control group of rats following an ordinary diet (*n* = 6); B) a group of rats to whose diet GSE was added (200 mg GSE/day) (*n* = 6); C) a group given cadmium (2 mg Cd/day—concretely, CdCl_2_ dissolved in water) (*n* = 6); and D) a group given a diet containing cadmium (2 mg Cd/day) to which GSE was added (200 mg GSE/day) (*n* = 6).

The extract was taken from the seeds of the Cerason grapevine variety; its preparation and characterization are described in Section 3.2. (*Grapevine Seed Extract*). The extract was weighed daily at a dose of 200 mg, before being administered to the experimental brown rats (200 mg GSE to each rat; 0.85 g/L kg of body mass). The GSE was administered with water.

The rats were given moist feed mixtures and harmless (infant) water ad libitum. Once per week, the animals had their bedding changed.

After 14 and 28 experimental days, three animals from each group were euthanized and their blood collected via a heart puncture. Before the collection, intraperitoneal medication was assigned with Fraxiparin (0.01 mL/kg) and, after that, the animals were given diethyl ether under inhalation anesthesia. A blood sample was then collected into heparin-coated micro test tubes (or tubes with EDTA—for analyses of MT and cadmium; without subsequent centrifugation), and this was followed by gentle centrifugation (800 g/10 min) and the collection of blood plasma for further analysis. The liver and right kidney were extracted for further analysis.

The study was conducted in accordance with the Declaration of Helsinki, and the protocol was approved by the Expert Commission for Ensuring the Welfare of Experimental Animals of Mendel University in Brno (for project IGA-ZF/2016-AP011).

### 3.2. Grapevine Seed Extract

Grapevine seeds (Cerason variety—Merlot × Seibel 13 666) were sorted, cleaned, dried, and ground. The sorting took place via the marcs on a separation machine, where the seeds were separated from the peels. The seeds separated in this way were subsequently rinsed, the empty seeds removed, and the remaining seeds cleaned. The cleaned and washed seeds were dried in a dryer at 50 °C for 12 h. This was followed by grinding of the seeds using an IKA MF 10 Basic laboratory grinder (IKA Werke, Staufen im Breisgau, Germany).

The ground seeds were extracted in 75% ethanol for 120 h (22 °C, shaken at 50 RPM; IKA KS 260 Basic, manufacturer: IKA Werke, Staufen im Breisgau, Germany) in a ratio of one part of ground seeds to ten parts of ethanol (*m/m*). After this, the extract was placed into 25 mL test tubes (Eppendorf, Hamburg, Germany) and centrifuged (Hettich MIKRO 220 Centrifuge; Hettich, Westphalia, Germany) at 4 °C and RCF 25,000× *g*. A centrifuged sample was taken from the test tube and transferred to a vacuum evaporator distillation flask (IKA RV 10 digital V-C; IKA Werke, Staufen im Breisgau, Germany), where the ethanol evaporated. The evaporation took place in a vacuum at 90 °C, and the extract was transferred to Petri dishes and lyophilised in a vacuum at −53 °C (Heto PowerDry PL 3000 lyophilizer; Trigon-plus, Čestlice, Czech Republic). Having been prepared, the extract was administered to the experimental animals.

### 3.3. Determination of Antioxidant Activity and Total Polyphenolics Content

In order to ensure the objectivity of the results, four fundamentally different methods were used to determine the antioxidant activity. Samples were analyzed on a BS-400 automatic spectrophotometer (Mindray, Shenzen, China). All samples were analyzed in triplicate, the resulting value corresponding to the average of these measurements. These analyzes were performed following Sochor et al. [35].

#### 3.3.1. Determination of Antioxidant Activity Using the ABTS Method

A solution for the determination of antioxidant activity by ABTS assay was prepared by mixing two solutions—solution 1: 7 mM solution of ABTS prepared by weighing m = 9.60 mg per 5 mL of distilled water; and solution 2: 4.95 mM solution of potassium persulfate prepared by weighing m = 1.67 mg per 5 mL of distilled water. The resulting solution was diluted 1:10 with distilled water and left in the dark and cold for 12 h.

Then, 150 µL of reagent R1 (7 mM ABTS and 4.95 mM potassium persulfate) was pipetted into plastic cuvettes, followed by the addition of 3 µL of the sample, and measured in a spectrophotometer (λ = 660 nm) for 12 min. According to the calibration curve, the absorbance was converted into the equivalent content of gallic acid; the antioxidant activity was calculated from the calibration curve using gallic acid as a standard (10–200 mg∙L^−1^), and the results were expressed as gallic acid equivalents.

#### 3.3.2. Determination of Antioxidant Activity Using the DPPH Method

First, m = 9.35 mg of radical DPPH was weighed. This amount was transferred to a 250 mL volumetric flask and made up with methanol.

Then, 150 µL of reagent R1 (0.095 mM DPPH) was pipetted into plastic cuvettes, followed by the addition of 15 µL of the sample to be measured. The DPPH test is based on the ability of the stable free radical 2,2-diphenyl-1-picrylhydrazyl to react with hydrogen donors. DPPH shows strong absorption in the ultraviolet-visible spectroscopy (UV-VIS) spectrum. Absorbance was measured for 12 min at λ = 505 nm. According to the calibration curve, the absorbance was converted to an equivalent gallic acid content.

#### 3.3.3. Determination of Antioxidant Activity Using the Ferric Reducing Antioxidant Power Method

Three solutions were used to determine the antioxidant activity using the ferric reducing antioxidant power (FRAP) method—(1) TPTZ solution: 10 mM TPTZ (m = 78.02 mg) dissolved in 25 mL of 40 mM HCl; (2) FeCl_3_ solution: 20 mM FeCl_3_ (m = 135.13 mg) dissolved in 25 mL of distilled water; and (3) acetate buffer solution: 0.02 M acetate buffer pH 3.6 (m = 775 mg sodium acetate dissolved in 250 mL of distilled water, pH adjusted with acetic acid). The three solutions were mixed in the ratio 1:1:10 (TPTZ: FeCl_3_: acetate buffer).

Then, 150 µL of reagent was pipetted into plastic cuvettes and 3 µL of the sample was added. Absorbance was measured for 12 min at λ = 605 nm. Antioxidant activity was calculated from the calibration curve using gallic acid as a standard (10–200 mg∙L^−1^). Results were expressed as gallic acid equivalent.

#### 3.3.4. Determination of Antioxidant Activity Using the Chlorophyllin Free Radical Method

A total of 200 µL of reagent R1 (0.1 M HCl, chlorophyll extract, reaction buffer, catalyst) was pipetted into plastic cuvettes and then 8 µL of the sample was added. This method uses the ability of chlorophyllin (sodium-copper salt of chlorophyll) to accept and donate electrons while stably changing the absorption maximum. This process is conditioned by the alkaline environment and the addition of a catalyst. Absorbance was measured for 12 min at λ = 450 nm. The last measurement values were used for the calculation. Results were expressed as gallic acid equivalent.

#### 3.3.5. Determination of Total Polyphenol Concentration

The Folin–Ciocalteu method was used to determine total polyphenolic compounds. All samples were analyzed in triplicate, and the resulting value was obtained as the average of these measurements.

A 40 µL sample was pipetted into a cuvette (3 mL) and diluted with 1960 µL of distilled water. Subsequently, 50 µL of Folin–Ciocalteu reagent was added to the cuvette. The mixture was shaken thoroughly and, after 3 min, 300 µL of 20% Na_2_CO_3_ decahydrate solution was added. The reaction mixture was shaken and incubated at 22 °C for 120 min. After this time, the absorbance (SPECORD 210; Carl-Zeiss, Jena, Germany) was measured at λ = 750 nm against a blank. Results were expressed as gallic acid equivalent.

### 3.4. Determination of Antioxidants by HPLC

Determination of Individual Antioxidant Components Using High-Performance Liquid Chromatography with Mass spectrometer.

Analyses of samples used for the estimation of flavonoids and proanthocyans were performed in an Agilent Technologies 1200 (Jet Stream Technologies, Santa Clara, CA, USA) chromatographic system consisting of the following devices: mobile phase reservoirs, degasser, binary pump, sample reservoir with an autosampler, and mass detector with an Agilent 6460 Triple Quadrupole LC/MS (Jet Stream Technologies, Santa Clara, CA, USA). Separation of samples was performed in Zorbax SB C18 chromatographic columns with dimensions 50 × 2.1 mm and the size of particles 2.7 µm.

The period of analysis was always 6 min. The flow rate of the carrier gas was 0.6 mL∙min^−1^. The organic component of the mobile phase was methanol gradient grade (solvent B) and the aqueous constituent contained 0.05 M of ammonium formate (solvent A). The gradient of the moving phase was as follows: time 0 min: 50% of solvent A and 50% of solvent B; time 2.5 min: 100% of solvent A and 0 % of solvent B; time 5 min: 50% of solvent A and 50% of solvent B. The temperature of the column thermostat was adjusted to 45 °C.

### 3.5. Determination of Selected Biochemical Markers

Biochemical markers in the blood, plasma, liver, and kidney of the animals were monitored to determine the protective effect of GSE against the negative effects of cadmium. The following analyses were performed:

(1) Plasma analyses: total plasma antioxidant activity, creatinine, urea, ALT, AST.
(2) Blood analyses: MT.
(3) Liver analyses: SOD, CAT, MT.
(4) Kidney analyses: MT.

#### 3.5.1. Determining the Total Plasma Antioxidant Activity, Creatinine, Urea, ALT, and AST

A BS-400 automated biochemical analyzer (Mindray, Shenzen, China) was used to analyze the total antioxidant activity of the plasma, creatinine, urea, ALT, and AST.

##### Determination of Total Antioxidant Activity of Plasma

The free radical method was used to determine the antioxidant activity of the plasma [35]. This method uses the ability of chlorophyllin (the sodium-copper salt of chlorophyll) to accept and donate electrons while stably changing the absorption maximum. This process is conditioned by the alkaline environment and the addition of a catalyst.

A total of 200 µL of reagent R1 (0.1 M HCl, chlorophyllin extract, reaction buffer, and catalyst) was pipetted into plastic cuvettes, after which 8 µL of the sample was added. The absorbance was measured for 12 min at λ = 450 nm, and the last measurement value was used for the calculation. Antioxidant activity was calculated from the calibration curve using gallic acid as a standard and the results were expressed as a gallic acid equivalent.

##### Determination of Creatinine Content

The principle of the method is the photometric determination of the color complex of creatinine with alkaline picrate, the rate of formation of which is proportional to the concentration of creatinine in the sample. A diagnostic kit from Greiner (Greiner GmbH, Pleidelsheim, Germany) was used to determine the creatinine.

A total of 180 µL of reagent R1 (0.16 mM sodium hydroxide) was pipetted into the cuvette, followed by the addition of 10 µL of the sample and 30 µL of the reagent R2 (4 mM picric acid). The absorbance was measured for six minutes at λ = 505 nm. The absorbance value of the reagent R1 alone with the sample and the absorbance value after 6 min incubation with reagent R2 were used for the calculation.

##### Determination of Alanine Aminotransferase

A diagnostic kit from Greiner (Greiner GmbH, Pleidelsheim, Germany) was used to determine ALT. First, 150 µL of reagent R1 (100 mM Tris buffer pH 7.5; 500 mM L-alanine, 1200 U/L lactate dehydrogenase) and 15 µL of plasma sample were automatically pipetted into the cuvettes. Subsequently, 30 µL of reagent R2 (15 mM 2-oxoglutarate, 0.18 mM NADH) was automatically pipetted and the mixture was incubated for 5 min. Absorbance was measured at λ = 340 nm. The absorbance values of reagent R1 alone with the sample and the absorbance values after 5 min incubation with the sample were used for the calculation. The result was converted according to the calibration curve to the enzyme activity in µkat/L.

##### Determination of Aspartate Aminotransferase

A diagnostic kit from Greiner (Greiner GmbH, Pleidelsheim, Germany) was used to determine AST. First, 150 µL of reagent R1 (80 mM Tris buffer pH 7.8, 240 mM L-aspartate, 1200 U/L malate dehydrogenase) and 15 µL of plasma sample of experimental animals were automatically pipetted into the cuvettes. Subsequently, mixing and incubation were performed at 37 °C for 270 s. Next, 30 µL of R2 reagent (15 mM 2-oxoglutarate, 0.18 mM NADH) was automatically pipetted and the mixture was incubated for 5 min. Absorbance was measured at λ = 340 nm. The absorbance values of reagent R1 alone with the sample and the absorbance values after 5 min incubation with the sample were used for the calculation. The result was converted according to the calibration curve to the enzyme activity in µkat/L.

##### Determination of Urea

The following procedure was used to determine the urea: 200 µL of reagent R1 (Tris buffer pH 7.8, ADP, urease, and GLDH) and 200 µL of reagent R2 (2-oxoglutarate, NADH) were automatically pipetted into cuvettes, followed by a sample of volume 4 µL, after which the mixture was mixed and incubated at 37 °C for 30 s. Subsequently, the absorbance at λ = 340 nm was measured and after another 60 s, the absorbance was measured again. The absorbance values after 30 s and after another 60 s were used for the calculation. The values were subtracted from each other and the result was converted according to the calibration curve to the urea content.

##### Determination of Total Proteins Using the Biuret Method

The determination of total proteins by the Biuret method is described in detail in another publication [9]. A total of 180 μL of Biuret reagent (15 mM sodium potassium tartrate, 100 mM NaI, 5 mM KI, and 5 mM CuSO_4_) was pipetted into the cuvette, followed by 45 μL of the sample. After ten minutes of incubation at 37 °C, the absorbance at wavelength λ = 546 nm was measured. The principle here is the reaction of the peptide bond with Cu (II) in an alkaline environment, with the formation of a blue-violet color. The absorbance value of the reagent alone and the absorbance value after ten minutes of incubation with the sample were used for the calculation.

#### 3.5.2. Determination of Superoxide Dismutase (SOD) and Catalase (CAT)

##### Determination of SOD Activity

A commercial kit (19160 SOD, Merck KGaA, Darmstadt, Germany) was used to determine SOD activity (SOD, EC 1.15.1.1). The measurement was performed in a plastic cuvette on a BS 400 automatic analyzer (Mindray, Shenzen, China). First, 200 µL of reagent R1 (20× buffer diluted WST solution) was pipetted into the cuvette and the reagent was incubated at 37 °C for 108 s (1 min, 48 s). Then, 20 µL of sample was pipetted and, at 378 s (6 min, 18 s), the reaction was started by adding 20 µL of reagent R2 (167× buffer diluted enzyme solution). The mixture was incubated for 72 s (1 min, 12 s) and then absorbance was measured at λ = 450 nm. The reaction kinetic was measured for 180 s (3 min) and the absorbance was read every nine seconds. The instrument calculated the average increase in absorbance per minute (ΔA) and the SOD activity of the measured samples, according to the calibration equation. The results were expressed in U/mL and subsequently converted to mg of protein.

##### Determination of CAT Activity

The determination of the CAT (following Aebi [36]) was performed on a Specord 210 instrument, a two-beam UV-VIS spectrophotometer (SPECORD 210; Carl-Zeiss, Jena, Germany), in quartz cuvettes (light path = 1 cm, cuvette volume 1 mL) at λ = 240 nm and at T = 25 °C. First, 950 µL of reagent (10 mM H_2_O_2_ in 50 mM phosphate buffer and 0.5 mM EDTA, pH 7.0) was pipetted into the cuvette, after which 50 µL of the sample was pipetted, the content of the cuvette mixed by pipetting, and the absorbance immediately recorded for 60 s at five-second intervals. The CAT activity was calculated according to the calibration equation:

$$U/\mathrm{mL}\;=\;\frac{\Delta A/\min.\;\mathrm{corrected}\;\mathrm{for}\;\mathrm{blank}\;\times \;\mathrm{sample}\;\mathrm{dilution}\;\times \;\mathrm{reaction}\;\mathrm{volume}\;\mathrm{in}\;\mathrm{mL}}{\mathrm{sample}\;\mathrm{volume}\;\mathrm{in}\;\mathrm{mL}\;\left(0.05\right)\;\times \;0.0436}$$

The results were expressed in U/mL and subsequently converted to mg of protein.

#### 3.5.3. Determination of Metallothionein Content

The determination of the metallothionein concentration in the blood, liver, and kidneys of the brown rats was performed using the adsorptive transfer stripping technique (AdTS) with differential pulse voltammetry (DPV). For the analysis, 200 µL of the analyzed supernatant was taken and mixed with 1 800 µL of Brdička’s reagent in a measuring vessel, after which the electrochemical determination itself was performed. The assay was performed in compliance with a previously described protocol [37]. A classic three-electrode connection was used. The working electrode was a hanging mercury drop electrode (HMDE) with a drop area of 0.4 mm^2^, while the reference electrode was Ag/AgCl/3 M KCl and the auxiliary was a platinum electrode. The base electrolyte (1 mmol·L^−1^ [CO(NH_3_)6] Cl_3_ and 1 mol·L^−1^ of ammonium buffer; NH_3_(aq) + NH_4_Cl, pH 9.6) was changed for each measured sample. The DPV parameters were as follows: initial potential 0.7 V, final potential 1.75 V, bubbling 5 s, deposition time 240 s, modulation time 0.05 s, interval time 0.25 s, potential step 0.00105 V, scan rate 0.0042 V/s, and modulation amplitude 0.02505 V.

The MT determination was performed on a measuring device consisting of a 695 Autosampler connected to a VA Stand 693 measuring unit and controlled by a 693 VA TraceAnalyser control unit (all Metrohm, Herisau, Switzerland). The entire measuring system was cooled to 4 °C using a Julabo F25 (Julabo, Seelbach, Germany). The obtained voltammograms were exported to the VA Database 2.2 program (Metrohm, Herisau, Switzerland), and the analyzed samples were deoxygenated by the active bubbling of argon with a purity of 99.999%.

#### 3.5.4. Determination of Cadmium Content in the Blood, Liver, and Kidney

##### Decomposition of Samples

An Ethos One microwave extractor (Milestone, Sorisole, Italy) was used for the decomposition of the liver and kidney samples, and a Multiwave 3000 Microwave Extractor (Anton Paar Graz, Austria) was used for the decomposition of the blood samples. Mineralization took place in a wet way, using 65% nitric acid (HNO_3_ suprapure) (Merck KGaA, Darmstadt, Germany) and 30% hydrogen peroxide (H_2_O_2_ suprapure) (Merck KGaA, Darmstadt, Germany).

A total of 0.4 g of liver or kidney sample was weighed into a series of Teflon containers, and 5 mL of 65% HNO_3_, 3 mL of 30% H_2_O_2_, and 2 mL of Milli Q H_2_O were added to the sample. The containers were closed and the instrument started according to the manufacturer’s instructions. The microwave decomposition lasted for 40 min at 210 °C. Subsequently, the samples were cooled for 45 min and stored at 4 °C in PE containers. 

A total of 0.01 g of a blood sample was weighed into a series of glass containers, and 300 µL of 65% HNO_3_ and 200 µL of 30% H_2_O_2_ were added to the sample. The containers were closed and the instrument started according to the manufacturer’s instructions. The microwave decomposition lasted for 40 min at a temperature of 140 °C. Subsequently, the samples were cooled for 45 min and stored at 4 °C until the determination by AAS.

##### Determination of Cadmium by Atomic Absorption Spectrometry

The cadmium was determined on an Agilent 280/240 Series AA Systems device (Agilent Technologies). The liver and kidney samples were analyzed in an acetylene–air flame (Fa AAS), and the blood samples by electrothermal atomization (ETA AAS) at a wavelength of 228.8 nm. A calibration series with concentrations of 0–20 µg·L^−1^ for analyses on ETA AAS and 0–10 mg·L^−1^ for Fa AAS was prepared from a standard solution of cadmium (Analytika Praha, Prag, Czech Republic), with a concentration of 1.000 ± 0.002 g·L^−1^. This was followed by a measurement of the calibration series solutions and a construction of the calibration curves, after which the mineralizates of the blood, liver, and kidney samples were measured.

### 3.6. Statistical Analysis

All measurements were performed in triplicate. Outlier values were identified with the Grubbs test and excluded (in total, two outliers were found). Before statistical analysis, the data were transformed by logarithmic transformation and analyzed by one-way ANOVA (each time point separately) with a Tukey HSD post-hoc test. The threshold for statistical significance was set as *p* < 0.05.

## 4. Conclusions

There has been a recent pharmaceutical and economic trend to look for new drugs among plant secondary metabolites, which are substances that can help to protect against a range of diseases. Thanks to the important biologically active components that grapevine seeds contain in their matrix, they can be (and already are) used for medical purposes. Grape seed extracts are known to protect tissues against oxidative stress-related damage caused by the toxic heavy metal cadmium. This protection is probably influenced by the phenolic composition of the used grape seeds. In this study, a grape seed extract prepared from the semi-resistant variety Cerason with a rather high phenolic content was tested for protective effects against cadmium-induced alterations. The studied GSE was able to increase plasma antioxidant activity when administered alone and lower cadmium content in the liver and kidneys of the rats when co-administered. Accordingly, the increases of hepatic marker enzyme ALT and kidney marker creatinine caused by cadmium were slowed down by GSE. Furthermore, the decreases in the activities of antioxidant enzymes SOD and CAT caused by cadmium were slowed down by GSE co-administration. These results show that GSE is a potent protective agent against the effects of heavy metals.

## Figures and Tables

**Table 1:** Antioxidant activity and total polyphenolic compound content of the extract from seeds of the Cerason variety.

DPPH^●^	FRAP	ABTS^●+^	FR	TP
12.6 ± 0.34	30.7 ± 0.42	15.4 ± 0.31	7.3 ± 0.15	9.3 ± 0.35

DPPH: 2,2-diphenyl-1-picrylhydrazyl, FRAP: ferric-reducing antioxidant power, ABTS: 2,2’-azino-bis (3-ethylbenzothiazoline-6-sulfonic acid), FR: free radicals, TP: total polyphenols. The results are expressed in mg/g gallic acid equivalent (GAE).

**Table 2:** Content of selected antioxidant components in grape seed extract.

AP	AG	HY	IS	KA	MY	QE	RU	A2	B1
3.02 ± 0.11	4.58 ± 0.23	221 ± 3.2	11.7 ± 0.38	0.21 ± 0.03	27.1 ± 0.47	7.85 ± 0.42	22.2 ± 0.71	359 ± 5.3	7.15 ± 0.21

Apigenin (AP), astragalin (AG), hyperoside (HY), isorhamnetin (IS), kaempferol (KA), myricetin (MY), quercitrin (QE), rutin (RU), procyanidin A2 (A2), procyanidin B1 (B1). Results are expressed in µg/g of dry matter.

## Data Availability

The data are archived at the Laboratory of Chemistry and Biochemistry in Mendel University in Brno, Faculty of Horticulture, Department of Viticulture and Enology, Valtická 337, 691 44 Lednice, Czech Republic.

## Sample Availability

Samples are not available from the authors.
